# Identification of a novel PANoptosis-associated prognostic signature and immune landscape analysis in neuroblastoma with experimental validation

**DOI:** 10.3389/fimmu.2026.1853357

**Published:** 2026-05-26

**Authors:** Yuanliang Tang, Zhibo Deng, Yun Zheng, Wanli Yu, Ming Cheng, Hong Xiao

**Affiliations:** 1 Neurosurgery Department, Two Rivers Hospital Affiliated to Chongqing Medical University, Chongqing, China; 2 Orthopedics department, the Second Affiliated Hospital of Chongqing Medical University, Chongqing, China; 3 Institute for Immunology and Pathogenesis, Chongqing Medical University, Chongqing, China; 4 Neurosurgery Department, Chongqing People's Hospital, Chongqing, China; 5 Department of Ophthalmology, Chongqing Youyoubaobei Women's and Children's Hospital, Chongqing, China

**Keywords:** immunoinfiltration, machine learning, neuroblastoma, PANoptosis, prognosis, WGCNA

## Abstract

**Background:**

Neuroblastoma (NB) is the most common extracranial pediatric solid tumor, and the prognosis of high-risk patients remains poor, while early diagnosis is still challenging. Although PANoptosis plays an important role in tumor development and progression, its role in NB has not been systematically investigated.

**Methods:**

GSE49710 (n = 498) was used as the training cohort, providing transcriptomic profiles and clinical annotations. Patients were stratified by Children’s Oncology Group (COG) risk classification. PANoptosis core genes were defined as the intersection between differentially expressed genes (DEGs) associated with COG risk and a curated PANoptosis gene set. Based on those core genes, consensus clustering analysis stratified patients into PANoptosis-related clusters (PANclusters). Differential gene analysis, survival analysis, and gene set variation analysis (GSVA) in PANclusters were conducted subsequently. Weighted gene co-expression network analysis (WGCNA) was applied to identify co-expression modules most strongly associated with PANclusters. Candidate genes were further subjected to Cox regression analysis and least absolute shrinkage and selection operator (LASSO) analyses to construct a prognostic model (NB index). The relationship between the NB index and the immune microenvironment and drug therapy was then evaluated, and a predictive nomogram was developed. External validation was performed using the TARGET cohort (n = 155) and an additional cross-platform cohort GSE62564 (n = 498). Finally, functional experiments were conducted to validate key genes.

**Results:**

Based on the PANoptosis gene set, GSE49710 was divided into two distinct clusters with different prognoses. In PANclusters, there were significant differences in cell cycle, DNA replication, mismatch repair, and regulation of autophagy. The yellow gene module in WGCNA was most associated with NB risk. 116 risk genes were obtained by intersecting the module genes with DEGs among patients in the PANcluster subgroup. Through Cox regression analysis and LASSO algorithm, an eight-gene signature was used to build the NB index. Patients with high NB index exhibited significantly worse survival, which was consistently validated in external cohorts. Notably, GSVA analysis demonstrated that PANoptosis activity was significantly decreased in the high-NB-index group, suggesting that suppression of PANoptosis may contribute to NB progression. The risk score was associated with immune-checkpoint expression and with predicted responses to immunotherapy and chemotherapy. Functional experiments showed that TOP2A knockdown inhibited proliferation, migration, and invasion of NB cells.

**Conclusions:**

We established an eight-gene PANoptosis-related prognostic index for neuroblastoma and highlighted four hub genes (KIF18A, EXO1, TOP2A, and TACC3) closely associated with NB risk and prognosis.

## 1 Introduction

Neuroblastoma (NB) is a malignant tumor that originates in neuroblastocytes and usually occurs in infants and adolescents (1). NB is clinically stratified using staging and risk-classification systems, including the International Neuroblastoma Staging System (INSS) and the Children’s Oncology Group (COG) risk classification (2, 3). Despite the availability of complex multimodal therapies, the prognosis of patients with high-risk NB is still poor, underscoring the need to elucidate disease mechanisms and find new treatments (4). The pathogenesis of NB is complex and involves many regulatory processes such as cell growth, proliferation and apoptosis (5). PANoptosis represents a novel type of programmed cell death (PCD) with distinctive features that incorporate elements of pyroptosis, apoptosis, and necroptosis (6). PANoptosis is primarily regulated by the PANoptosome complex, which is influenced by internal and external signals (7). PANoptosis contributes to tissue homeostasis and inflammatory regulation. Dysregulated PANoptosis may therefore influence NB initiation and progression, although its role in NB remains poorly characterized.

In NB, suppression of PCD is a key mechanism underlying abnormal tumor cell proliferation and survival. This phenomenon can be achieved through a variety of mechanisms (8). NB cells can inhibit the normal apoptotic signaling pathway through mutated or abnormally expressed genes, such as MYCN gene amplification, thereby impairing apoptotic capacity. In addition, overexpression of intracellular anti-apoptotic proteins (e.g. the Bcl-2 family) also leads to inhibition of apoptosis, thereby increasing the survival rate of tumor cells (9). Interactions between cytokines, growth factors, and tumor cells in the tumor microenvironment can affect the survival and proliferation of tumor cells. These factors may affect the physiological state of cells by regulating apoptosis-related signaling pathways, such as PI3K/Akt, MAPK, thereby promoting tumor cell survival. Pyroptosis is mediated by a class of proteases called inflammatory caspases (such as caspase-1 and caspase-4/5/11), which trigger inflammatory cell death under specific conditions (10). The development of NB is closely related to the surrounding inflammatory microenvironment. Inflammatory cells, such as macrophages and other immune cells, can release pro-inflammatory factors, such as interleukin-1β (IL-1β) and tumor necrosis factor-α (TNF-α), which can activate the inflammation-related cell death pathway. Other studies suggest that activation or inhibition of necroptosis related signaling pathways, such as RIPK1-RIPK3-MLKL pathway, may affect the survival and therapeutic response of NB cells (11, 12). Collectively, these studies suggest that each form of PCD involved in PANoptosis is associated with NB biological behavior and the tumor microenvironment. Therefore, we hypothesize that NB progression is closely associated with the integrated cell death regulatory network defined by PANoptosis.

PANoptosis-related gene signatures have shown prognostic value in other malignancies. For example, Zhang et al. identified PANoptosis-related genes that predict diagnosis and prognosis in hepatocellular carcinoma (13), and Jiang et al. developed a PANoptosis-based risk model for clear cell renal cell carcinoma with potential therapeutic implications (14). However, the prognostic and biological relevance of PANoptosis-related genes in NB has not been systematically evaluated. Therefore, a systematic investigation of PANoptosis in NB may provide novel insights into disease mechanisms and facilitate the development of more effective therapeutic strategies.

## 2 Methods

### 2.1 Data collection

Gene expression profiles and corresponding clinical data for neuroblastoma were obtained from the Gene Expression Omnibus (GEO) database (GSE49710, n = 498), which served as the training cohort. This dataset included 176 COG high-risk patients and 322 COG low-risk patients. An independent validation cohort was obtained from the Therapeutically Applicable Research to Generate Effective Treatments (TARGET) database. After excluding five samples lacking survival information, a total of 155 patients were included in the external validation cohort. In addition, an extra cross-platform validation cohort (GSE62564, n = 498), consisting of the same patient population as GSE49710 but generated using a different sequencing platform, was included to further assess the robustness and generalizability of the prognostic model. All gene expression data and corresponding clinical information were publicly available and obtained from the respective databases. Therefore, no additional ethical approval was required.

### 2.2 Differential expression analysis

Patients were divided into high- and low-risk groups according to COG classification, and differentially expressed genes (DEGs) between the two groups were identified. DEGs were identified using the R package “limma” with thresholds of |log2 fold change| > 1 and adjusted P< 0.05 (Benjamini–Hochberg correction). The DEGs were visualized using a volcano plot.

### 2.3 Gene Ontology and Kyoto Encyclopedia of Genes and Genomes enrichment analyses

The intersection between COG risk–associated DEGs and the PANoptosis gene set was defined as PANoptosis-related genes in NB. These genes were subjected to GO and KEGG enrichment analyses using the SangerBox platform (http://SangerBox.com/Tool). Adjusted P< 0.05 was considered statistically significant.

### 2.4 Consensus clustering analysis

Consensus clustering was performed using the ConsensusClusterPlus R package based on the expression profiles of PANoptosis-related genes in the 498 patients. The optimal number of clusters (k) was determined using the cumulative distribution function (CDF) curve and consensus matrices. The parameters were set as follows: reps = 50, pItem = 0.8, pFeature = 1, clusterAlg = “km”, distance = “euclidean”, and seed = 123. Principal component analysis (PCA) was conducted using the prcomp function in R to visualize cluster separation. Overall survival between clusters was compared using Kaplan-Meier (KM) curves and the log-rank test. DEGs between PANoptosis-related clusters (PANclusters) were identified using the “limma” R package with thresholds of |log2 fold change| > 1 and adjusted P< 0.05 (Benjamini–Hochberg correction). Gene sets (including c5.go.v7.4.symbols.gmt and c2.cp.kegg.v7.4.symbols.gmt) were obtained from the Molecular Signatures Database (MSigDB). GSVA enrichment analysis was conducted using the “GSVA” R package, and the results were visualized using the “pheatmap” R package.

### 2.5 Construction of protein–protein interaction network

A protein–protein interaction (PPI) network was constructed using the STRING database for candidate PANoptosis-related DEGs (PRDs), with a minimum required interaction score of 0.4. The PPI network was visualized using Cytoscape software (version 3.9.1) (16). The Molecular Complex Detection (MCODE) plugin in Cytoscape was used with default parameters to identify significant modules within the PPI network, and hub genes were subsequently extracted from the key modules.

### 2.6 Weighted gene co-expression network analyses

WGCNA was performed using the WGCNA R package on the GSE49710 expression data. Sample clustering was performed to detect and remove outlier samples. The soft-thresholding power (β) was determined to construct a scale-free network. The topological overlap matrix (TOM) was then created from the adjacency matrix. Genes were hierarchically clustered based on TOM dissimilarity to identify co-expression modules. Module–trait relationships between co-expression modules and PANclusters were evaluated. Genes from the module most significantly associated with PANclusters were intersected with PANcluster-related DEGs to identify candidate PANoptosis-associated genes.

### 2.7 Predictive model construction

Univariate Cox regression analysis, multivariable Cox regression, and least absolute shrinkage and selection operator (LASSO) regression analyses were performed using the survival, survminer, and glmnet R packages to select prognostic genes and construct the risk model. Eight PRDs were screened and a prognostic model was established. The following formula was used to calculate the individual NB risk score: $\text{Risk score}=\sum_{i=1}^{8}\beta i*Expi$, where *βi*, and *Exp_i_* represent the regression coefficient, and gene expression value, respectively. Patients were divided into high- and low-NB-index groups based on the median risk score. Differences in overall survival between groups of high- and low-NB index were evaluated using KM analysis and the log-rank test. The predictive accuracy of the model was assessed through performing the area under the curve (AUC) of the receiver operating characteristic (ROC) curve. The nomogram of NB patients was constructed according to the NB risk model, and its predictive accuracy was evaluated using calibration curves. The prognostic value of the NB risk model was validated in the independent TARGET cohort. Consistent results were observed in the GSE62564 cohort, further supporting the robustness of the model across different platforms.

### 2.8 Gene set variation analysis

To quantify PANoptosis activity at the sample level, GSVA was performed using the “GSVA” R package. The PANoptosis gene set was defined based on the curated gene list used in this study, and only genes present in the expression matrix were included for analysis. GSVA enrichment scores were calculated for each sample using the Gaussian kernel, which is appropriate for normalized transcriptomic data. The resulting GSVA score was defined as the PANoptosis score. To investigate the relationship between PANoptosis activity and the prognostic model, Spearman correlation analysis was performed between the PANoptosis score and the NB index. In addition, patients were stratified into high- and low-NB-index groups based on the median NB index, and differences in PANoptosis scores between groups were assessed using the Wilcoxon rank-sum test. All statistical analyses were conducted in R, and p< 0.05 was considered statistically significant.

### 2.9 Assessment of immune microenvironment and prediction of therapeutic sensitivity

We used Cell-type Identification by Estimating Relative Subsets of RNA Transcripts (CIBERSORT) to evaluate the degree of immune infiltration in different NB-index risk groups (17). Differences in immune cell infiltration between high- and low-NB-index groups (stratified by the median NB index) were evaluated using the Wilcoxon rank-sum test. Spearman correlation analysis was performed to assess the associations between the expression levels of the eight prognostic genes and immune cell infiltration. The activity of 14 cancer-related pathways was inferred using the “PROGENy” R package (18). Drug sensitivity was predicted based on the Genomics of Drug Sensitivity in Cancer (GDSC) database using the “oncoPredict” R package. The half-maximal inhibitory concentration (IC_50_) of each drug was estimated for each sample using “oncopredict” R package (19).

### 2.10 Cell culture and cell transfection

Human neuroblastoma cells SH-SY5Y (RRID: CVCL_0019) were purchased from HyCyte (Soochow, China) and cells were cultured according to the supplier’s instructions. Transfection of small interfering RNAs into SH-SY5Y cells was performed according to the Operation Handbook of the Lipofectamine^®^3000 reagent (Thermo Fisher Scientific). The siRNA sequences are listed in **Table 1**.

**Table 1:** Sequences of siRNAs.

Name	Nucleotide sequence	
Si-TOP2A - 1	Sense	5’-GGUCAGAAGAGCAUAUGAUTT-3’
	Antisense	5’-AUCAUAUGCUGUUCUGACCTT-3’
Si-TOP2A - 2	Sense	5’-GACCAACCUUCAACTAUCUTT-3’
	Antisense	5’-AGAUAGUUGAAGGUUGGUCTT-3’
Si-TOP2A - 3	Sense	5’-GGUGAGAUGUCACUAAUGATT-3’
	Antisense	5’-UCAUUAGUGACAUCUCACCTT-3’
Negative control (NC)	Sense	5’-UUCUCCGAACGUGUCACGUTT-3’
	Antisense	5’-ACGUGACACGUUCGGAGAATT-3’

### 2.11 EdU proliferation assay

The 10 μM 5-ethynyl-2′-deoxyuridine (EdU) medium was prepared according to the guidelines provided by the manufacturer (C10310-1, RiboBio, China). siRNA-transfected SH-SY5Y cells were seeded into 24-well plates. After the transfected cells reached the appropriate confluence, the medium was replaced with 100 μL of EdU medium and incubated at 37 °C with 5% CO_2_ for 2h. Cells were then fixed in 4% paraformaldehyde for 20 min and subjected to a 30-minute incubation with Apollo^®^ reagent (100 μL) at room temperature (RT). Afterwards, the cells were stained with DAPI dye and examined using a fluorescence microscope. The ratio of EdU-positive cells to the total number of DAPI-positive cells was calculated to determine cell proliferation.

### 2.12 RNA extraction and qRT-PCR methods

Total RNA was harvested from the transfected cells utilizing TRIzol reagent (Invitrogen, United States). With the use of the Evo M-MLV Mix Kit with gDNA Clean for qPCR (Accurate Biology, AG11728, China), the extracted total RNA was reverse-transcribed into cDNA. SYBR Green Premix Pro Taq HS qPCR Kit (Accurate Biology, AG11701, China) was used for the amplification. GAPDH was used as an internal control for mRNA qPCR. The data were processed using the 2^-ΔΔCt^ method. The primer sequences of the studied genes are as follows: TOP2A, forward 5’ – CTTAATACCGATCTACATGGCCA - 3’ and reverse 5’ – CTGCTTCCACATTGCTGCTG - 3’; GAPDH, forward 5’ – GGAGCGAGATCCCTCCAAAAT - 3’ and reverse: 5’ – GGCTGTTGTCATACTTCTCATGG - 3’.

### 2.13 Colony formation assays

The transfected cells were evenly seeded into a 6-well plate at a density of 1,000 cells per well. The cells were then cultured for 14 days, with medium refreshing and cell observation every 2 days. After that, the cells were washed once with PBS, fixed with 4% paraformaldehyde for 15 min, and stained with 1% crystal violet dye to visualize colonies.

### 2.14 Wound-healing assay

The transfected cells were cultured in 6-well plates until evenly spread, and the wells were delineated with a 10 μ pipette tip, followed by starvation culture in serum-free medium. Photographs were taken at 0, 24 h, and 48 h with a microscope and subsequently analyzed using ImageJ software.

### 2.15 Cell counting kit-8 assay

Cell viability was measured using cell counting kit-8 (C0038, Dojindo, Japan) according to manufacturer’s instructions. The transfected cells were inoculated into 96-well plates at a density of 4 × 10^3^/well, 10 μL CCK-8 solution was added to each well at 0,24,48,72 h (0 hour was defined as 8 hours after cell seeding), and the plates were returned to the incubator for 1 h. The absorbance of each well was measured at 450 nm using an enzyme-labeled instrument.

### 2.16 Transwell assay

The transfected cells (2 × 10^4^/ml) were inoculated into Transwell chambers (FALCON, USA) containing 250 μL serum-free medium, and 500 μL serum-containing medium was added to the bottom of the chambers. After 48 h, the chambers were fixed with 4% paraformaldehyde and stained with crystal violet. Five randomly selected fields of view were photographed under a microscope and cell counting was performed using Image J.

### 2.17 Statistical methods

All statistical analyses were performed using R software (version 4.1). Differences between two groups were assessed using the Wilcoxon rank-sum test unless otherwise specified. Correlations were evaluated using Spearman correlation analysis. Survival differences were analyzed using KM curves and the log-rank test. All tests were two-sided, and p< 0.05 was considered statistically significant.

## 3 Results

### 3.1 Identification and functional analysis of PANoptosis-related differentially expressed genes for NB

**Figure 1:**
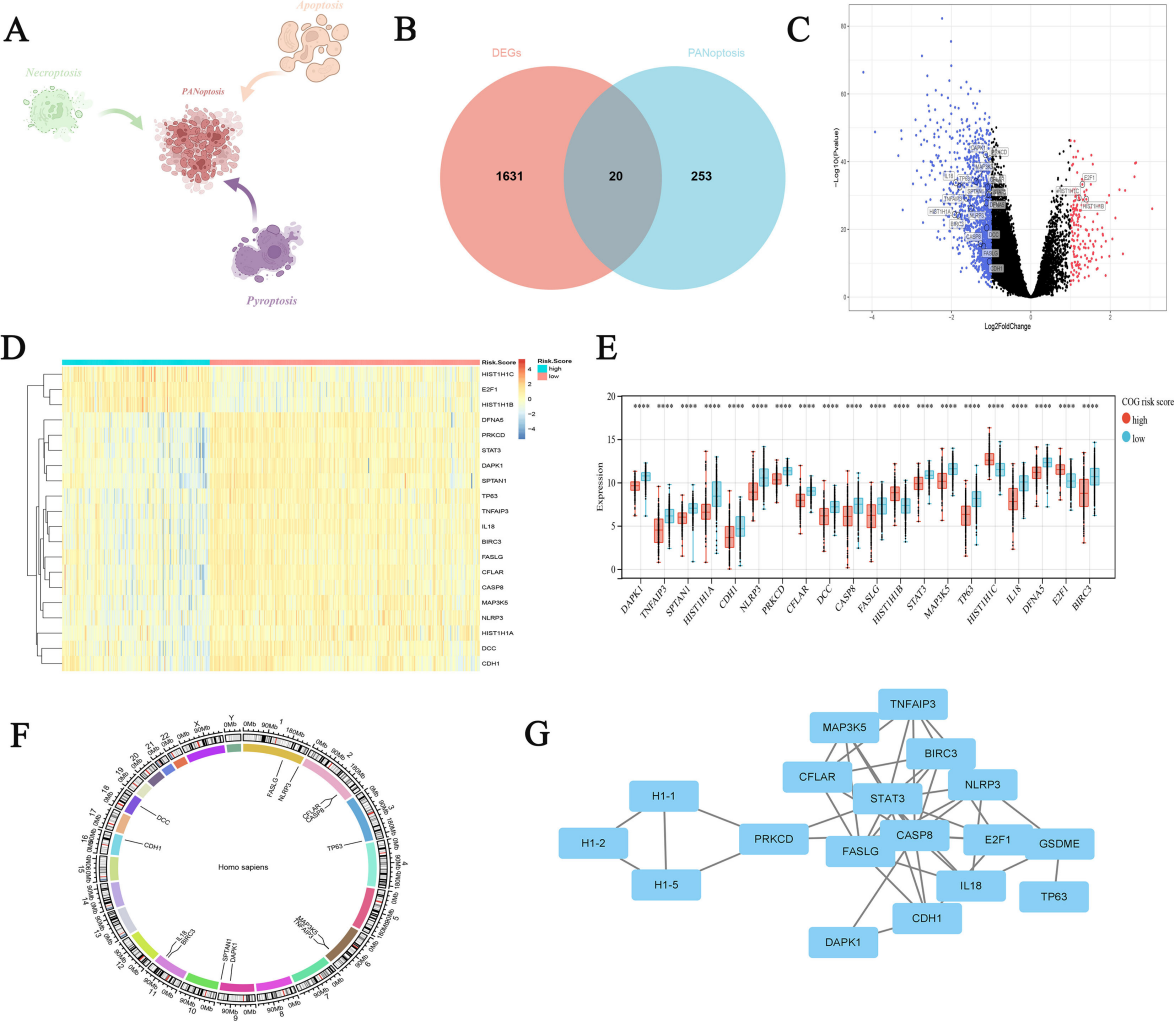
Identification of PANoptosis-related DEGs (PRDs) for NB. **(A)** Schematic diagram of the apoptotic mechanism (created in BioRender.com). **(B)** Venn diagram to identify 20 PRDs in NB patients from the GSE49710 data set. **(C)** Volcano plot of the 20 PRDs in the DEGs from GSE49710. **(D)** Heatmap of the 20 PRDs in GSE49710. **(E)** Expression difference of the 20 PRDs between COG high and low risk patients. **(F)** Locations of the 20 PRDs on 23 chromosomes. **(G)** PPI network of the 20 PRDs. An interaction score > 0.4 was set as significant differences and isolated nodes were removed. ****p< 0.0001.

**Figure 2:**
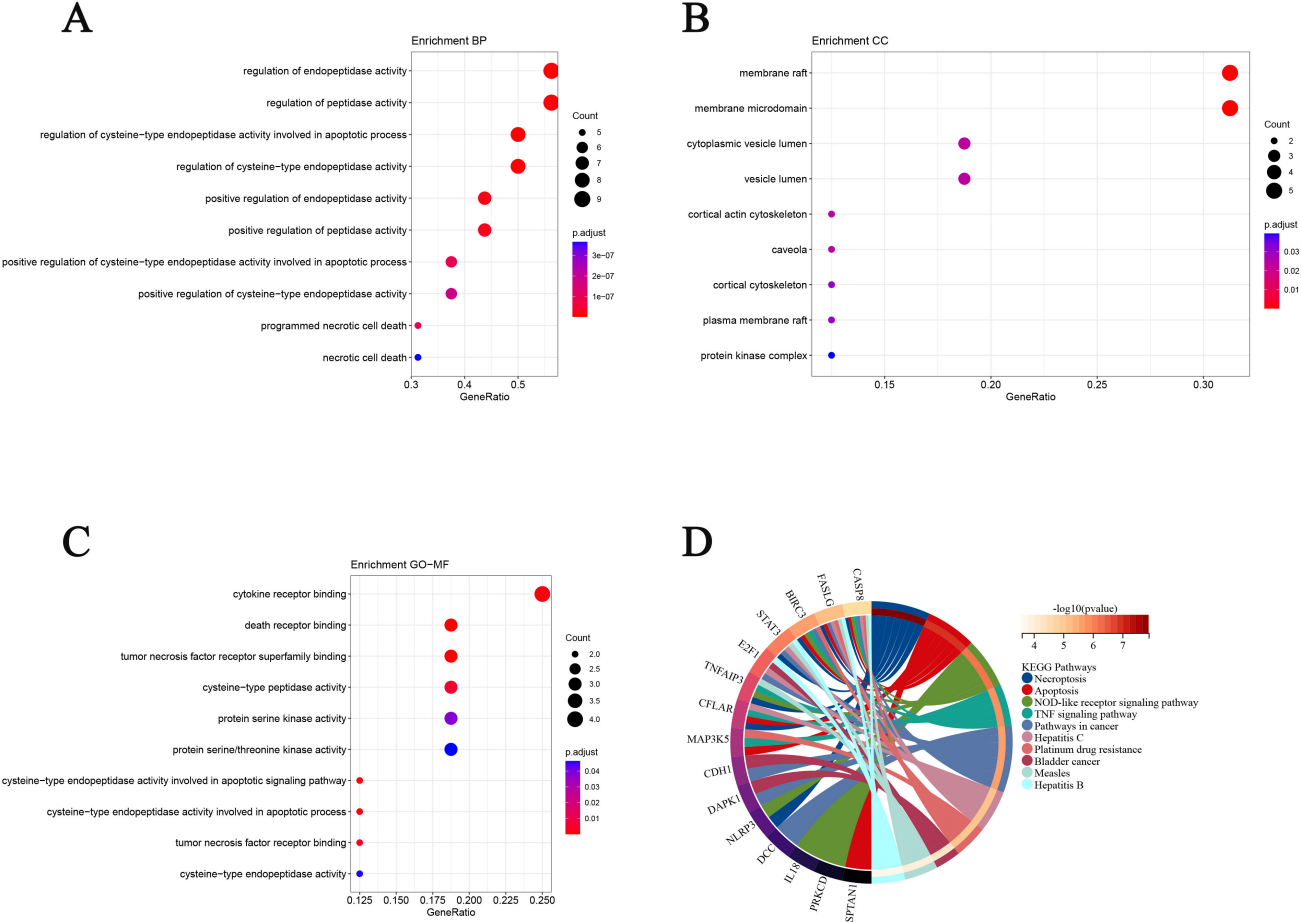
GO and KEGG enrichment analysis of the PRDs. **(A–C)** GO enrichment analyses based on the PRDs; **(D)** KEGG enrichment analyses based on the PRDs.

Referring to previous literature (20, 21), the PANoptosis gene set consisted of 273 genes from pyroptosis, apoptosis, and necroptosis (**Figure 1A**). Intersecting DEGs between COG high-risk and low-risk groups in GSE49710 with the PANoptosis gene set identified 20 PRDs (**Figure 1B**). These 20 PRDs were visualized using a volcano plot (**Figure 1C**), and their expression patterns across NB samples are shown in a heatmap (**Figure 1D**). The expression of these 20 PRDs was significantly different between the two groups (**Figure 1E**). The chromosome location map revealed the genomic distribution of the PRDs across different chromosomes (**Figure 1F**). The PPI network illustrated close interactions among PRDs *(***Figure 1G***)*. We studied the biological function and related signaling pathways of PRDs by GO and KEGG enrichment analysis. Biological process (BP) analysis revealed that PRDs were mainly enriched in regulation of endopeptidase activity, regulation of peptidase activity and regulation of cysteine-type endopeptidase activity involved in apoptotic process (**Figure 2A**). Cell composition (CC) analysis showed that PRDs were mainly enriched in membrane raft, membrane microdomain and cytoplasmic vesicle lumen (**Figure 2B**). Molecular function (MF) analysis showed that PRDs were mainly enriched in cytokine receptor binding, death receptor binding and tumor necrosis factor receptor superfamily binding (**Figure 2C**). In addition, the KEGG enrichment analysis suggested that PRDs were mainly enriched in Necroptosis, Apoptosis, NOD-like receptor signaling pathway and TNF signaling pathway (**Figure 2D**).

### 3.2 PANoptosis related DEGs expression profiling identifies two PANoptosis subtypes with distinct prognoses

**Figure 3:**
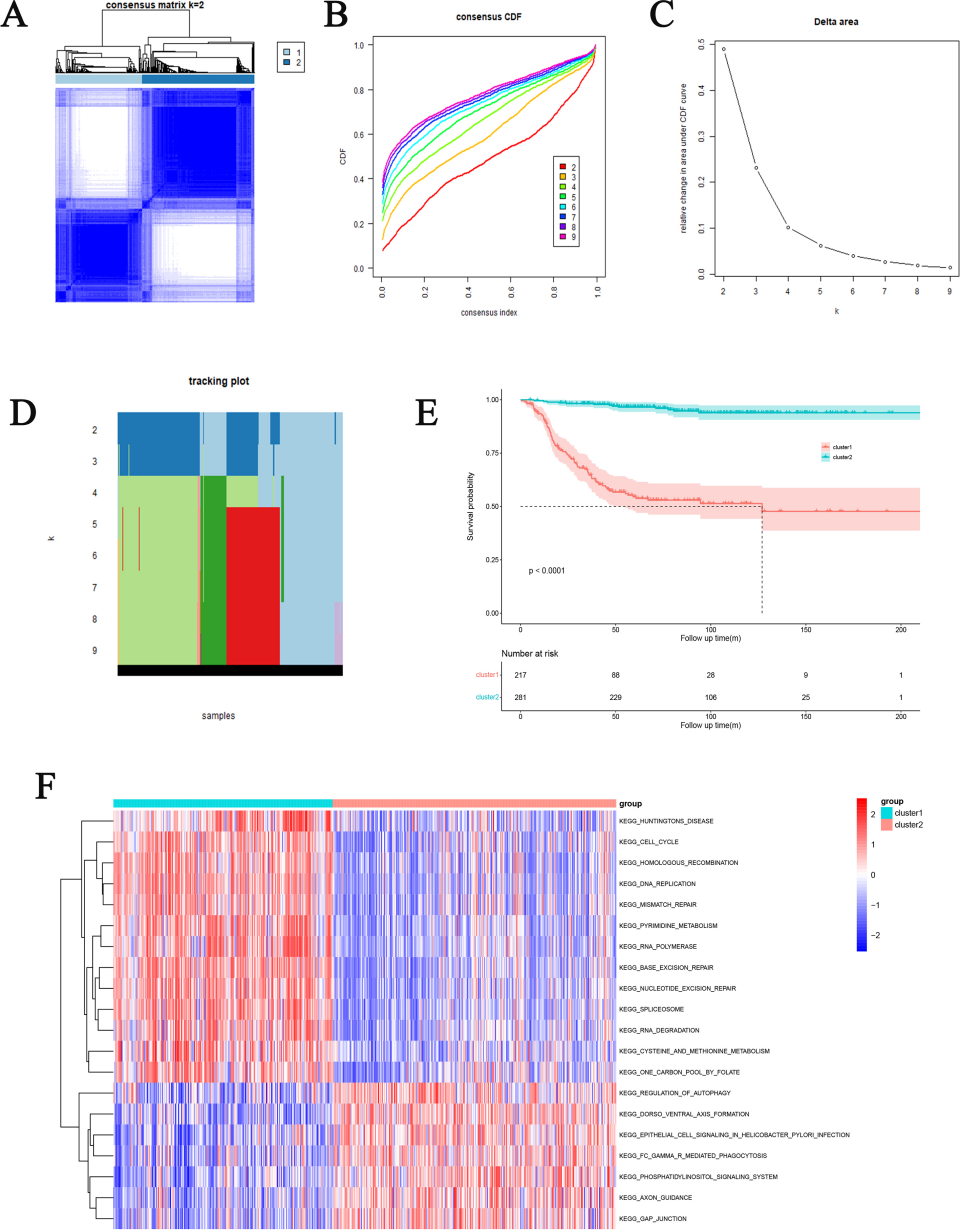
Two NB-PANoptosis subtypes with different prognoses were identified based on PRDs expression profiles. **(A)** GSE49710 was divided into two NB subtypes by consensus clustering. **(B)** Assessment of area under the CDF curve line when k = 2 to 9. **(C)** Tracking plot. The rows are samples, and the columns are k values. **(D)** Delta area plot. **(E)** KM survival analysis between two subtypes of PRDs. **(F)** Difference in the enriched pathways between two PANclusters.

We used consensus clustering analysis to group NB patients based on the expression profiles of 20 PRDs. Based on the CDF curves and consensus matrices, k = 2 was identified as the optimal number of clusters *(***Figures 3A–D***)*. We named the PANoptosis related clusters (PANclusters) Cluster1 (n = 217) and Cluster2 (n = 281). Survival analysis showed that the two PANclusters exhibited different clinical prognostic outcomes (**Figure 3E**), with Cluster1 having a lower overall survival rate (P<0.0001). Compared with Cluster2, Cluster1 had significant enrichment in cell cycle, homologous recombination, DNA replication and mismatch repair. In Cluster2, the enrichment of pathways such as regulation of autophagy, dorsoventral axis formation and phosphatidylinositol signaling system was more significant (**Figure 3F**).

### 3.3 WGCNA unveils modules linked to PANoptosis related clusters and NB risk

**Figure 4:**
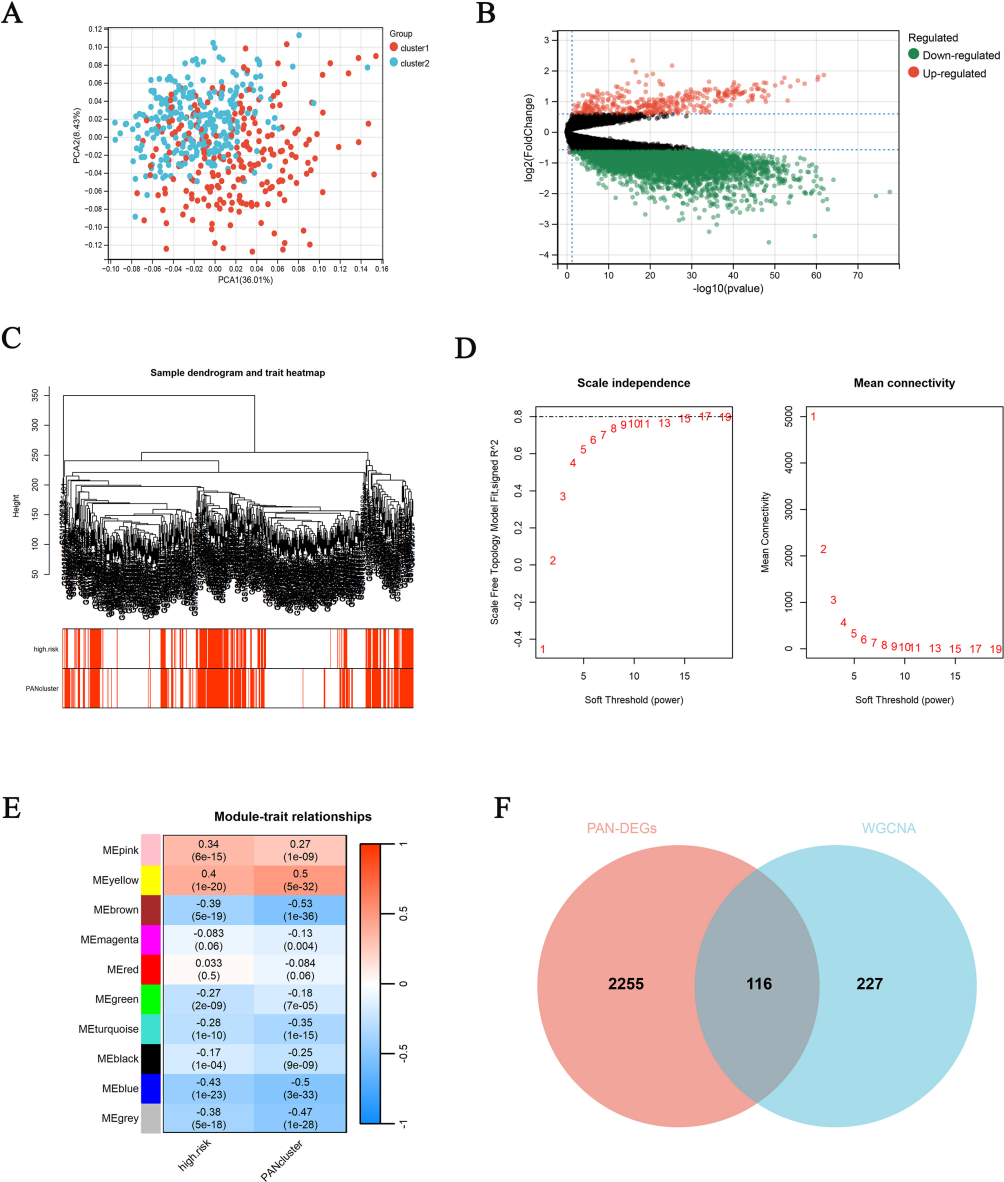
WGCNA co-expression network of PANclusters and selection of PANoptosis related feature genes (PRFGs). **(A)** PCA plot of two PANclusters. **(B)** Volcano plot of the DEGs from PANclusters. **(C)** Clustering dendrogram of PANcluters based on Euclidean distance. **(D)** Scale Independence and mean connectivity at different power values. **(E)** Relationships between PANclusters and 10 modules. Yellow module was most closely related to PANclusters in NB. **(F)** The Venn plot of 116 PRFGs in Yellow module and DEGs from PANclusters.

PCA demonstrated clear separation between PANclusters, indicating satisfactory clustering performance (**Figure 4A**). A total of 2371 DEGs were obtained for Cluster1 and Cluster2 (**Figure 4B**). Further, WGCNA was used to screen out key genes that may play an important role in PANclusters. The remaining samples were clustered after outliers were eliminated (**Figure 4C**). The soft-thresholding power was set to 15, at which the scale-free topology fit index reached 0.81, indicating an approximately scale-free network (**Figure 4D**). Based on the constructed network, genes were classified into 10 co-expression modules (**Figure 4E**). Among them, MEyellow module was the module with the highest correlation with PANclusters and COG risk (correlation index=-0.5, P< 0.0001). Finally, 116 PANoptosis related feature genes (PRFGs) were obtained by the intersection of PANclusters DEGs and MEyellow (**Figure 4F**).

### 3.4 Construction of PANoptosis prognostic model with multiple machine learning algorithms

**Figure 5:**
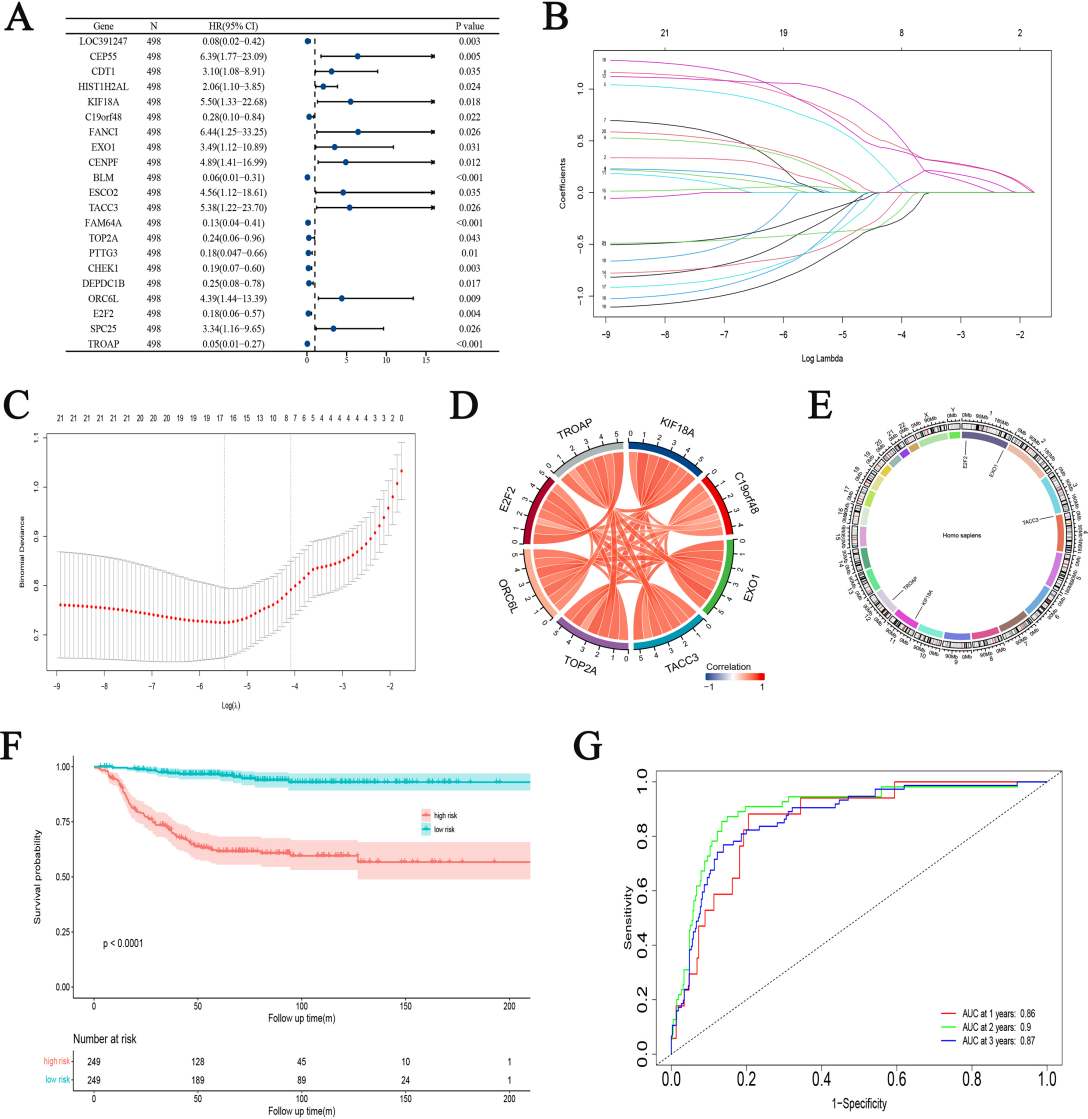
Construction of PANoptosis-related model in patients. **(A)** Multivariable Cox analysis of PANcluster related feature genes (PRFGs) in the GSE49710 dataset. **(B, C)** LASSO regression analysis and partial likelihood deviance on the prognostic genes. **(D)** Circle plot of the correlation between the eight genes obtained by Lasso regression analysis. **(E)** The location of genes with chromosomal information on the chromosome. **(F)** KM survival analysis between NB high and low risk index groups. **(G)** Time-dependent ROC curve of the NB index in the GSE49710.


Among 116 genes, univariate COX regression analysis excluded 95 variables. Multivariate Cox regression showed that all 21 remaining genes were significantly associated with OS (**Figure 5A**). LASSO Cox regression was subsequently applied for feature selection. As the penalty parameter λ increased, the number of retained genes decreased, and the optimal model was obtained at λ.min. Ultimately, eight genes with the highest prognostic value were selected (**Figures 5B, C**). The eight genes (KIF18A, C19orf48, EXO1, TACC3, TOP2A, E2F2, TROAP, ORC6L) were incorporated into the prognostic signature, and a protein–protein interaction network illustrated their close associations (**Figure 5D**). Excluding genes without chromosomal information, the chromosomal mapping showed that E2F2 and EXO1 were located on chromosome 1 (**Figure 5E**). Based on the coefficients of the eight genes, the NB-index risk score was calculated by the following formula:

$$\begin{array}{c} \text{NB index risk score}=(0.106\times \text{the expression of }KIF18A)+(0.058\times \text{the expression of }C19orf48)+(0.452\times \text{the}\\ \text{expression of }EX01)+(0.566\times \text{the expression of }TACC3)+(-0.06\times \text{the expression of }TOP2A)+(-0.278\times \text{the}\\ \text{expression of }E2F2)+(-0.20\times \text{the expression of }TROAP)+(0.409\times \text{the expression of }ORC6L) \end{array}$$

We used the NB-index score to divide the training set of patients into an NB-high risk group and an NB-low risk group (high 249 vs Low 249 patients). KM analysis showed that the prognosis of low-risk group was significantly better than that of high-risk group (P<0.0001, **Figure 5F**). In addition, we evaluated the accuracy of the AUC of the NB index as a predictive model, and the results demonstrated strong predictive performance of the NB-index model for 1-, 3-, and 5-year survival.

### 3.5 Validation of PANoptosis prognostic model

**Figure 6:**
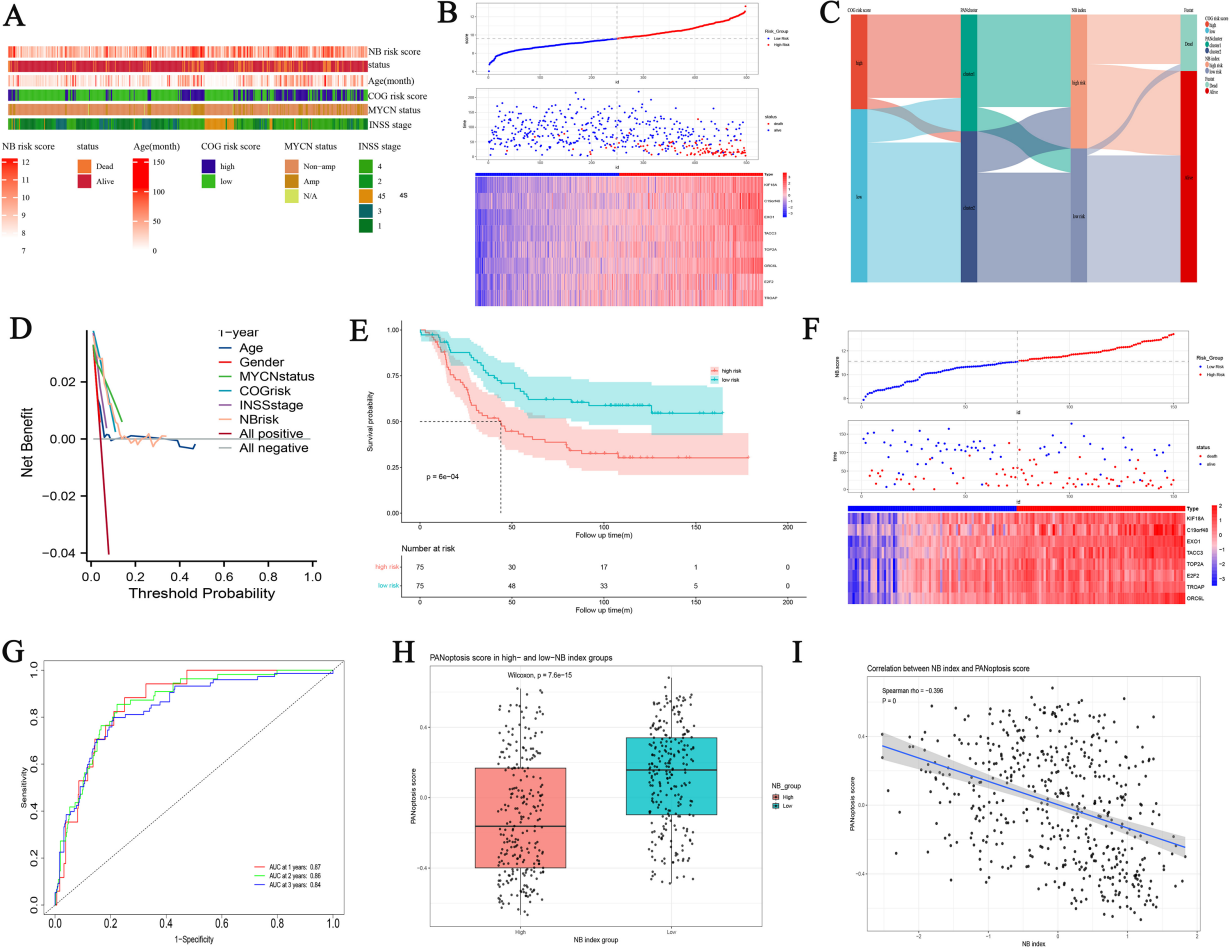
Prognostic assessment of NB index model in NB. **(A)** Distribution of INSS stage, MYCN status, COG risk, age at diagnosis, OS status, and NB risk scores in training set. **(B)** The distribution of NB risk scores, the association of NB risk scores and OS, and the 8 PRFGs expression. **(C)** Sankey diagram for COG risk score, PANclusters, NB index and OS. **(D)** The DCA curves of the NB risk score, INSS stage, COG risk, MYCN status, Gender, and age at 1 year. **(E)** KM survival analysis between NB high and low risk index groups in test set from TARGET database. **(F)** The distribution of NB risk scores, the association of NB risk scores and OS, and the 8 PRFGs expression in test set. **(G)** ROC curves of the model for predicting the 1-, 3-, and 5-year survival in GSE62564. **(H)** The PANoptosis scores from the high- and low-NB index group of GSE62564. **(I)** The correlation between the NB index and PANoptosis score from GSE62564.

Late INSS, MYCN amplification, and age greater than 18 months were highly associated with poor prognosis for NB (22). **Figure 6A** depicted the correlation between these characteristics and the predicted NB risk score. The results showed that the NB high risk score was associated with poor clinical indicators such as INSS stage 4, MYCN amplification, older age, and mortality. We further investigated the relationship between patient prognosis, gene expression, and the NB index, and observed a significant decline in survival as the NB index increased (**Figure 6B**). As expected, eight PRFGs were risk factors, and their expression levels rose progressively with higher NB-index scores. We used Sankey diagram to visualize the relationship between the NB-index risk group and clinical features, and the results showed that the COG-high-risk group mainly converged to Cluster1 and NB-high-risk group, and was associated with poor prognosis (**Figure 6C**). It is worth mentioning that from the area under the DCA curve (**Figure 6D**), it can be clearly seen that the prediction ability of NB-index exceeds that of COG risk and INSS stage, indicating the superior prediction ability of this model.

To test the robustness of the NB index model as a predictive model, we validated the model using the TARGET database (validation set). Applying KM analysis, we observed a significant decrease in patient survival with the increase of the NB index (**Figure 6E**). As in the training set, the expression of eight PRFGs was upregulated with the increase of NB index (**Figure 6F**). In conclusion, the NB index model showed significant predictive value for patient survival in both the training set and the validation set, and the higher the NB index score, the worse the prognosis.

To further evaluate the robustness and cross-platform applicability of the NB index model, we validated the model in an independent cohort GSE62564 (n = 498), which contains the same patient population as the training cohort but was generated using a different sequencing platform. Consistent with the training dataset, patients in the high-NB-index group exhibited significantly worse overall survival compared with those in the low-NB-index group (P< 0.0001), confirming the stability of the model across platforms. Time-dependent ROC analysis demonstrated that the NB index maintained strong predictive performance, with AUC values of 0.87, 0.86, and 0.84 for 1-, 3-, and 5-year survival, respectively (**Figure 6G**). Furthermore, GSVA analysis revealed that PANoptosis activity was significantly decreased in the high-NB-index group compared to the low-NB-index group (Wilcoxon test, P< 0.001) (**Figure 6H**), which was consistent with the findings in the training cohort. Spearman correlation analysis further demonstrated a significant negative correlation between the NB index and PANoptosis score (Spearman rho = −0.396, P< 0.001), suggesting that higher risk scores are associated with reduced PANoptosis activity (**Figure 6I**). Although the eight-gene signature is not composed of canonical PANoptosis executors, these findings indicate that the NB index is closely associated with PANoptosis pathway activity. Collectively, these results support the robustness, reproducibility, and biological relevance of the NB index model across different transcriptomic platforms.

### 3.6 Construction of a nomogram for predicting survival in NB

**Figure 7:**
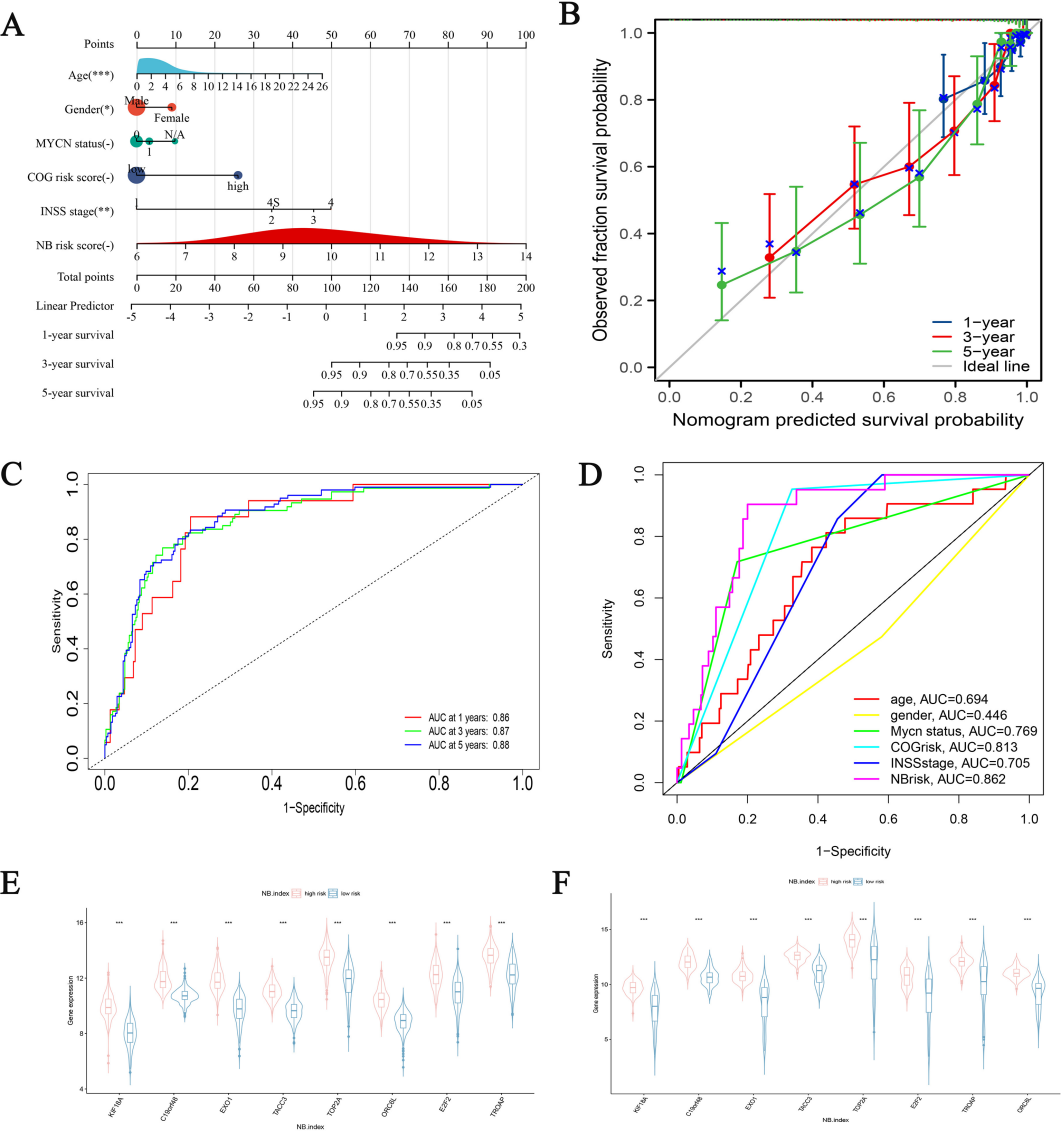
Construction of a nomogram and the evaluation of its effects. **(A)** The nomogram predicting the risk for NB patients with clinical characteristics. **(B)** Calibration graphs showed that the actual survival rates of NB patients were close to the nomogram-predicted survival rates. **(C)** ROC curves of the model for predicting the 1-, 3-, and 5-year survival. **(D)** 1-year ROC curves for each characteristic in the nomogram. **(E)** Violin diagram of 8 PRFGs in training set. **(F)** Violin diagram of 8 PRFGs in text set. **p< 0.01, ***p< 0.001.

To better predict survival in patients with NB, we constructed a nomogram integrating a prognostic model of NB with key clinical factors, including age, sex, MYCN amplification status, COG risk score, and INSS stage. Each item was scored according to the actual situation of the patient, and the cumulative score was then used to predict 1-, 3-, and 5-year survival (**Figure 7A**). Subsequently, the 1-, 3- and 5-year prediction accuracy of the constructed nomogram was verified, as shown in the calibration curve (**Figure 7B**), and the observed values in the model fit well with the optimized values. The AUC of 1-, 3-, and 5-year OS in the ROC curve were 0.86, 0.87, and 0.88, respectively (**Figure 7C**). In addition, the ROC curves of clinical characteristic in nomogram were plotted (**Figure 7D**), and the AUC of NB-index, MYCN amplification status, COG risk score and INSS stage were all greater than 0.70. Notably, the predictive power of NB-index exceeds MYCN amplification status, COG risk score and INSS stage. Besides, the violin diagram showed that the eight PRFGs of the constructed model differed significantly in both the training set and the validation set (**Figures 7E, F**).

### 3.7 Immune infiltration and sensitivity to treatment in two risk subgroups

**Figure 8:**
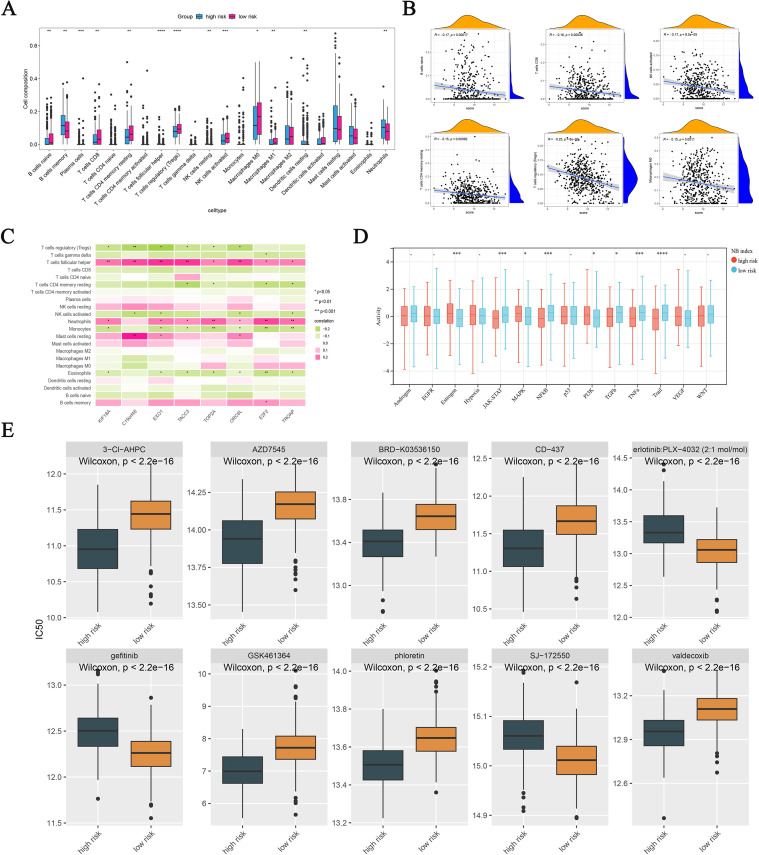
Immune infiltration and sensitivity to chemotherapy. **(A)** Analyzing the immune cell infiltration levels of NB samples from high and low NB index groups by CIBERSORT algorithm. **(B)** Relevance between risk score and different immune cell types. **(C)** Relevance between immune cells and eight PRFGs. **(D)** Boxplot of tumor-related pathways activity. **(E)** Significant IC_50_ difference from top 10 therapeutic drugs between two NB risk subgroups. *p< 0.05, **p< 0.01, ***p< 0.001, ****p< 0.0001.

Previous studies have shown that tumor microenvironment (TME) is closely related to the metastasis of tumor cells (23), so understanding the TME characteristics of PANoptosis-related subtypes is important for immunotherapy of NB-related subtypes with poor prognosis. CIBERSORT algorithm was used to analyze the levels of immune cell infiltration in different NB-index risk groups. The results showed that the expression of memory B cells, plasma cells, follicular helper T cells, and neutrophils was higher in the subtypes with NB-index high risk group. Naïve B cells, CD8^+^ T cells, resting memory CD4^+^ T cells, regulatory T cells, activated NK cells, and M0/M1 macrophages were lower in abundance in the high-risk group (**Figure 8A**). As shown in **Figures 8B**, NB-index scores were inversely correlated with naïve B cells, CD8^+^ T cells, activated NK cells, resting memory CD4^+^ T cells, regulatory T cells, and M0 macrophages. We also found that these eight potential PRFGs were closely related to the abundance of many immune cells (**Figure 8C**). As shown in **Figure 8D**, there were significant differences in the activity of 8 of the 14 tumor-related pathways between the high-risk and low-risk groups of the NB index. Specifically, estrogen signaling, MAPK, and PI3K pathways activity was significantly higher in the high-risk group than in the low-risk group. In contrast, the JAK–STAT, NF-κB, TGF-β, TNF-α, and TRAIL pathways showed higher activity in the low-risk group. According to the calculation results of GDSC database, we predicted whether the high- and low-risk group of NB-index had different sensitivities to 545 anticancer drugs. The ten chemotherapy drugs with the highest difference in drug sensitivity were 3-CI-AHPC, AZD7545, BRD-K03536150, CD-437, erlotinib, PLX-4032, gefitinib, GSK461364, phloretin, and SJ-172550 (**Figure 8E**). These results suggested that most chemotherapy drugs may have better therapeutic effects on NB in the low-risk group of NB-index, while PLX-4032, gefitinib and SJ-172550 may have potential targeted therapeutic effects on the high-risk group of NB-index.

### 3.8 Evaluation of the PANoptosis related feature genes by RT-qPCR

**Figure 9:**
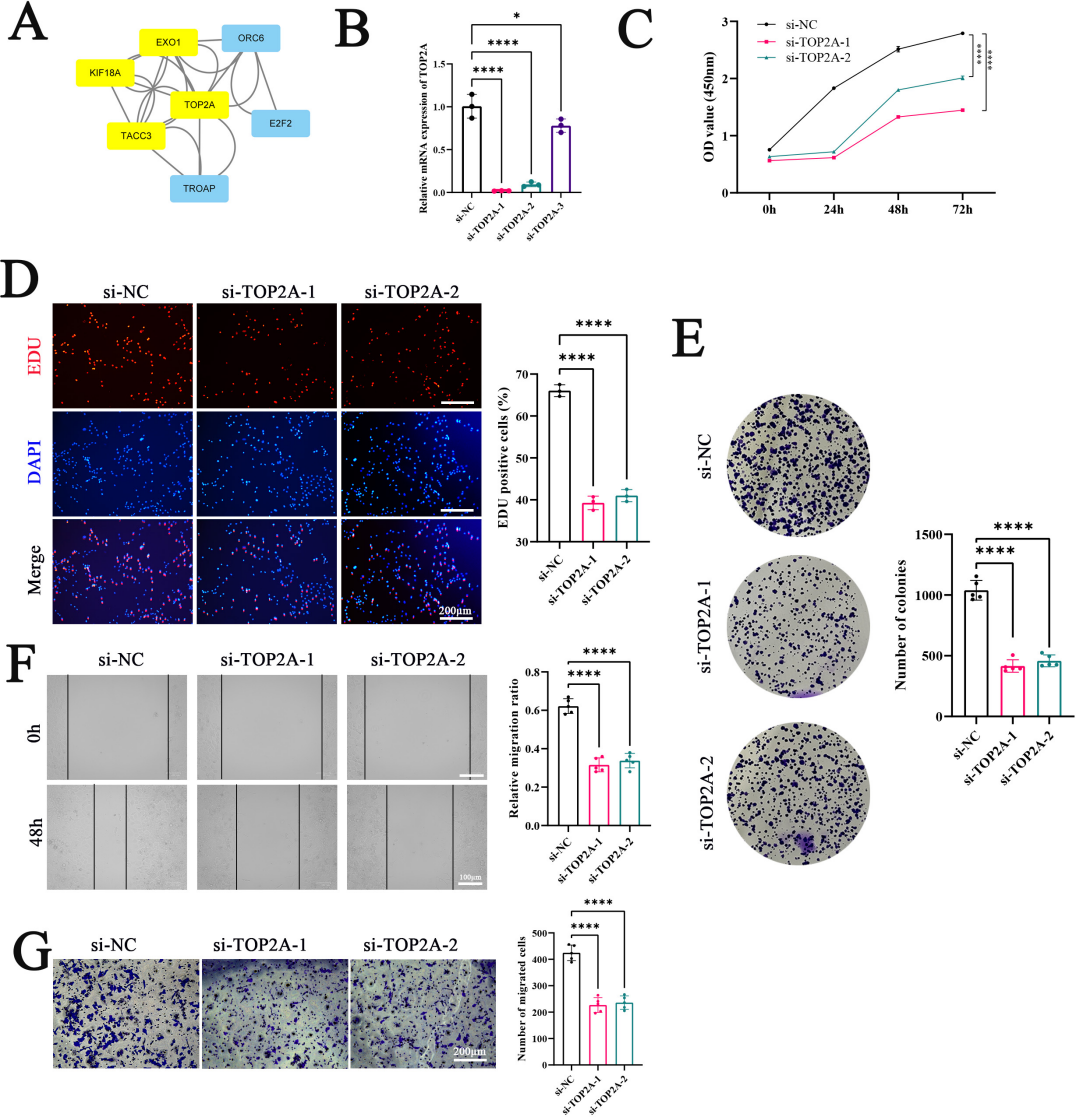
Evaluation of the hub PRFGs by RT-qPCR. **(A)** The MCODE algorithm was used to find the hub PRFGs in PPI network. **(B)** RT-qPCR analysis showed the inhibitory efficiency of TOP2A in SH-SY5Y cells. Cell Viability **(C)** and Proliferation **(D, E)** of SH-SY5Y After TOP2A Inhibition Assessed by CCK-8 and EdU or Colony formation Assays. **(F)** Conduct wound healing tests to evaluate the migration ability of SH-SY5Y cells after TOP2A inhibition. **(G)** Transwell assay confirmed the decreased invasive ability of SH-SY5Y cells after the knockdown of TOP2A. ****p<0.0001.

The interactions among the eight genes included in the prognostic signature were evaluated by constructing a protein–protein interaction (PPI) network. To further identify key genes with central roles in the network, the MCODE algorithm was applied, and four highly connected hub genes (KIF18A, EXO1, TOP2A, and TACC3) were identified (**Figure 9A**). Among the four identified hub genes (KIF18A, EXO1, TOP2A, and TACC3), TOP2A was selected for subsequent functional validation based on multiple considerations. Specifically, TOP2A exhibited a central position in the PPI network with high connectivity, demonstrated strong prognostic significance in the Cox and LASSO regression analyses, and has been previously implicated in neuroblastoma progression and cell proliferation. Therefore, TOP2A was considered the most representative candidate for experimental validation in this study. To further verify the function of TOP2A, we synthesized three different siRNAs (si-TOP2A-1, si-TOP2A-2, and si-TOP2A-3) targeting TOP2A and transfected them into SH-SY5Y cells. The knockdown efficiency was detected by RT-qPCR *(***Figure 9B**). The results showed that all three sequences could effectively inhibit the expression of TOP2A, but si-TOP2A-1 and si-TOP2A-2 had more significant inhibitory effects. Therefore, we chose si-TOP2A-1 and si-TOP2A-2 for the subsequent experiments. CCK-8 and EdU proliferation assays showed that down-regulation of TOP2A inhibited the proliferation of SH-SY5Y cells (**Figures 9C, D**). Subsequent experiments, including plate colony formation assay (**Figure 9E**), scratch assay (**Figure 9F**), and Transwell assay (**Figure 9G**), consistently showed that SH-SY5Y cell migration, colony formation, and invasion ability were significantly reduced after TOP2A knockdown.

## 4 Discussion

PANoptosis is a new type of PCD, proposed in 2019 by Malireddi et al. (24). This form of PCD is considered an inflammatory process and is unique in that it combines elements of pyroptosis, apoptosis, and necroptosis, producing biological effects that cannot be explained by any of these processes alone (25). In recent years, the research of PANoptosis mainly focuses on chronic diseases, cancers, infectious diseases related to immune response (26–28). NB, a neuroendocrine tumor that usually develops in the adrenal glands, is the most common extracranial solid tumor in children (29). The survival rate for high-risk NB is only about 50%, and survivors are also subject to long-term chemotherapy (30). Although various new therapies have shown positive results in cell lines and mouse models (31), the distress caused by NB is still not fully resolved, indicating that our understanding of NB is not complete (32). Inducing PANoptosis may activate a broad inflammatory response, kill multiple cancer cells, stimulate durable immune protection, and enhance T-cell function and persistence in the TME (15). Simultaneously, PANoptosis is a key protective mechanism for cells when exposed to various stresses and injuries. Therefore, activating PANoptosis may make cancer cells more susceptible to treatment, thereby improving the therapeutic effect (33). PRDs are reported as prognostic markers in thyroid cancer (15), head and neck squamous cell carcinoma (34) and glioma (35) and other tumors. However, there is limited research on the potential biomarkers of the PANoptosis gene in NB. To our knowledge, this is the first study to explore the prognostic effects of PRFGs in NB. We not only constructed a prognostic model using the eight PRFGs, but also identified four hub genes as biomarkers for NB.

Based on 498 samples from GSE49710, a prognostic model of NB survival named NB index was established using eight PRFGs. The eight PRFGs are KIF18A, C19orf48, EXO1, TACC3, TOP2A, E2F2, TROAP, and ORC6L. Specifically, a nomogram model was constructed using NB index, age, sex, MYCN amplification status, COG risk score, and INSS stage, which has a stable predictive power of 1-, 3-, and 5-year survival probabilities. Therefore, this model may become a viable tool for predicting the survival probability of patients with NB. Meanwhile, each gene in the model showed significant differences between the high- and low-NB-index groups. These findings were consistently validated in the external TARGET cohort. Moreover, cross-platform validation in the GSE62564 dataset further confirmed the robustness and reproducibility of the NB index. Importantly, GSVA analysis revealed that PANoptosis activity was significantly reduced in the high-NB-index group and was inversely correlated with the NB index, suggesting that high-risk tumors may exhibit a PANoptosis-suppressed biological state. Furthermore, a PPI network constructed from the eight-gene signature identified four hub genes (KIF18A, EXO1, TOP2A, and TACC3) using the MCODE algorithm. These hub genes may occupy central positions within the interaction network and are likely involved in key biological processes associated with tumor progression and PANoptosis-related regulation. Meanwhile, the experimental results showed that SH-SY5Y cell migration, colony formation, and invasion ability were significantly reduced after TOP2A knockout.

KIF18A is a member of the kinesin superfamily and works as a master regulator of chromosome aggregation and centromere movements. In previous studies, KIF18A was identified as a potential therapeutic target for human breast cancer and low-grade gliomas (36, 37). Luo et al. (38) also believed that KIF18A may promote the proliferation, invasion and metastasis of liver cancer cells by promoting cell cycle signaling, Akt signaling, and MMP-7/MMP-9 related signaling pathway. Chen et al. (39) found that KIF18A, as a novel apoptosis-related gene, can predict the prognosis of patients with hepatocellular carcinoma.

EXO1 is an exonuclease associated with DNA mismatch repair, DNA double-strand break repair, nucleotide excision repair, and immunoglobulin maturation (40). Liu et al. found that EXO1 is a potential prognostic target for head and neck squamous cell carcinoma (41). Zhou et al. also found that EXO1 can predict the prognosis of lung adenocarcinoma based on apoptosis (42). It is also associated with a poor prognosis for prostate and breast cancer (43). Consistent with previous studies, EXO1 may also be a potential biomarker for MYCN-positive NB, both markers of poor prognosis.

TOP2A has been implicated in relieving transcription-induced supercoiling at highly active promoters (44). High expression of TOP2A is associated with shorter survival and poorer prognosis of NB. TOP2A can also form complexes with N-MYC and thereby participate in the progression of NB (45). TOP2A, a DNA topoisomerase II alpha enzyme, has also been shown to be one of the therapeutic targets of cancer drug (46).

TACC3 is the most oncogenic member of the transforming acidic coiled-coil domain-containing protein (TACC) (47). TACC3, as a gene associated with cell aging, has been shown to promote the proliferation of colorectal cancer cells and subjected with high expression present an immunosuppressive microenvironment (48). Targeting TACC3 can induce cancer cells death during mitosis and interphase, making it a promising approach for treating malignancies (49). Consistent with our findings, these four PRFGs are also shown to be potential biomarkers for the diagnosis and treatment of NB malignancies.

Considering that TME plays an important role in anti-tumor immunity, we studied the relationship between NB-index risk score and immune cell abundance, and found that five cell types were positively correlated and 7 cell types were negatively correlated. In previous reports, TACC3, EXO1, and TOP2A are three PRFGs that are closely related to immune cells and have been identified as novel indicators of tumor prognosis and immunotherapy response through the immune microenvironment (48, 50–52). The results are consistent with most of our findings, suggesting that all four PRFGs have the potential to predict the prognosis of NB. In clear cell renal cell carcinoma, TOP2A is associated with CD8+ T cells and plays a key role in the metastasis process (53). The results of CIBERSORT showed that naive B cells, activated NK cells, and resting CD4 memory T cells were negatively correlated with NB index scores (P< 0.001). Naive B cells have been reported to secrete cytokines that hinder cancer cell proliferation (54). Enhancing the abundance of NK cells and T cells can improve TME, which is associated with better tumor prognosis (55). From a mechanistic perspective, the observed association between the NB index and immune infiltration may be partly explained by the suppression of PANoptosis activity in high-risk tumors. Reduced PANoptosis may lead to decreased inflammatory signaling and impaired immune cell recruitment, thereby contributing to an immunosuppressive tumor microenvironment. In addition, the altered activity of key signaling pathways such as MAPK, PI3K, and JAK–STAT observed in high-risk groups may further promote tumor progression and immune evasion. These findings suggest that PANoptosis-related regulatory networks may play a critical role in shaping the tumor immune landscape in neuroblastoma. Therefore, the anti-tumor treatment effect of NB-index low-risk group is better than that of the high-risk group. IC_50_ results also showed that the low-risk subgroup was more sensitive to a greater number of drugs, and the effects of immunotherapy and chemotherapy were echoed by TME analysis. Therefore, our findings are helpful to further understand the effect of PANoptosis on NB, and may provide a reference for immunotherapy and chemotherapy regimen for NB patients. The high expression of KIF18A, EXO1, TOP2A and TACC3 in NB tissues further suggests that these four PRFGs may be potential biomarkers for the diagnosis and treatment of NB.

There are several limitations in this study. Firstly, the data analyzed are all from public databases. Secondly, limited molecular biology experiments have only provided the preliminary verification, and no further functional experiments have been performed. Although we identified four hub genes, only TOP2A was subjected to functional validation in this study due to resource and experimental constraints. The lack of experimental validation for the remaining hub genes (KIF18A, EXO1, and TACC3) represents a limitation and warrants further investigation in future studies. Further clinical samples and more detailed clinical features are needed to comprehensively evaluate our findings.

## 5 Conclusions

In conclusion, by combining bioinformatics and molecular biology experiments, we validated that PANoptosis is strongly associated with NB-related survival and immune regulation, and identified four PRFGs, especially TOP2A, as potential biomarkers for diagnosis and therapy. This study provides a deeper understanding of PANoptosis in NB and proposes potential strategies for targeted therapy of NB.

## Data Availability

The original contributions presented in the study are included in the article/supplementary material. Further inquiries can be directed to the corresponding authors.
